# The Molecular Signature of HIV-1-Associated Lipomatosis Reveals Differential Involvement of Brown and Beige/Brite Adipocyte Cell Lineages

**DOI:** 10.1371/journal.pone.0136571

**Published:** 2015-08-25

**Authors:** Rubén Cereijo, José Miguel Gallego-Escuredo, Ricardo Moure, Joan Villarroya, Joan Carles Domingo, Joan Fontdevila, Esteban Martínez, Maria del Mar Gutiérrez, María Gracia Mateo, Marta Giralt, Pere Domingo, Francesc Villarroya

**Affiliations:** 1 Department of Biochemistry and Molecular Biology, Institute of Biomedicine of the University of Barcelona, and CIBER Fisiopatología de la Obesidad y Nutrición, Barcelona, Catalonia, Spain; 2 Infectious Disease Unit, Hospital de la Santa Creu i Sant Pau, Barcelona, Spain, and Red de Investigación en SIDA, Instituto de Salud Carlos III, Spain; 3 Department of Plastic Surgery, Hospital Clínic de Barcelona, Barcelona, Catalonia, Spain; 4 Infectious Diseases Unit, Faculty of Medicine, University of Barcelona, Hospital Clinic of Barcelona (HCB), Barcelona, Spain; GDC, GERMANY

## Abstract

Highly active antiretroviral therapy has remarkably improved quality of life of HIV-1-infected patients. However, this treatment has been associated with the so-called lipodystrophic syndrome, which conveys a number of adverse metabolic effects and morphological alterations. Among them, lipoatrophy of subcutaneous fat in certain anatomical areas and hypertrophy of visceral depots are the most common. Less frequently, lipomatous enlargements of subcutaneous fat at distinct anatomic areas occur. Lipomatous adipose tissue in the dorso-cervical area (“buffalo hump”) has been associated with a partial white-to-brown phenotype transition and with increased cell proliferation, but, to date, lipomatous enlargements arising in other parts of the body have not been characterized. In order to establish the main molecular events associated with the appearance of lipomatosis in HIV-1 patients, we analyzed biopsies of lipomatous tissue from “buffalo hump” and from other anatomical areas in patients, in comparison with healthy subcutaneous adipose tissue, using a marker gene expression approach. Both buffalo-hump and non-buffalo-hump lipomatous adipose tissues exhibited similar patterns of non-compromised adipogenesis, unaltered inflammation, non-fibrotic phenotype and proliferative activity. Shorter telomere length, prelamin A accumulation and SA-β-Gal induction, reminiscent of adipocyte senescence, were also common to both types of lipomatous tissues. Buffalo hump biopsies showed expression of marker genes of brown adipose tissue (e.g. UCP1) and, specifically, of “classical” brown adipocytes (e.g. ZIC1) but not of beige/brite adipocytes. No such brown fat-related gene expression occurred in lipomatous tissues at other anatomical sites. In conclusion, buffalo hump and other subcutaneous adipose tissue enlargements from HIV-1-infected patients share a similar lipomatous character. However, a distorted induction of white-to-“classical brown adipocyte” phenotype appears unique of dorso-cervical lipomatosis. Thus, the insults caused by HIV-1 viral infection and/or antiretroviral therapy leading to lipomatosis are acting in a location- and adipocyte lineage-dependent manner.

## Introduction

HIV-1 infection is currently treated using highly active anti-retroviral therapy (HAART), which has dramatically improved patients’ life expectancy and quality. However, long-term HAART in HIV-1 patients is associated with a number of adverse effects, including a form of lipodystrophy designated HAART-associated lipodystrophy syndrome (HALS). Although removal of the most toxic components of HAART cocktails has decreased the incidence of HALS in recent years, the syndrome remains a frequent concern.

Altered adipose tissue distribution is a hallmark of HALS, with patients often showing lipoatrophy of subcutaneous adipose tissue in the face, arms, legs and buttocks; however, hypertrophy of visceral adipose tissue similar to abdominal obesity is also common [1,2]. Less frequently, lipomatous alterations take place in HAART-treated patients, the most common of which is a dorso-cervical accumulation, commonly referred to as “buffalo hump” (BH) [3]. Another site of prevalent lipomatous adipose enlargement is the pubic area [4], and a pattern of accumulation of fat leading to circumferential enlargement of the neck, also called “bullfrog neck”, has also been reported [5]. In addition, multiple reports have indicated the presence of lipomas in HIV-1–treated patients scattered at different anatomical areas, such as the neck, trunk, and limbs [4,6].

To date, molecular characterization of lipomatous tissue in HIV-1-infected patients has been limited to the dorso-cervical BH fat. These studies have revealed a decrease in mitochondrial DNA (mtDNA) abundance in BH. However, this alteration is not likely to account for the hypertrophic response of the dorso-cervical fat because other subcutaneous areas, which exhibit the opposite lipoatrophic behavior, show similar levels of mtDNA depletion. Adipogenesis and inflammation, however, are not altered in BH; this is in contrast to the decreased expression of adipogenesis marker genes and induction of pro-inflammatory genes in lipoatrophic areas of the same patients [7]. Several studies have shown that adipose tissues in BH biopsies express uncoupling protein-1 (UCP1) [8,9] or 5’-deiodinase [10]—markers of brown adipose tissue (BAT)—especially in samples obtained from substantially enlarged lipomatous tissue requiring surgery.

With the recent use of positron emission tomography (PET) has come the recognition that BAT in adult humans is present mainly in the cervical-supraclavicular region [11–14]. However, lipomatous BH appears not to be composed of fully functional brown adipocytes, and a pathologically distorted adipocyte differentiation process that results in incomplete transition from the white to brown adipocyte phenotype has been suggested. Among the observed changes in BH cells, the acquisition of a highly proliferative status confirms the lipomatous features of fat accumulation in BH [7,15].

Recent years have seen important advancements in our understanding of brown adipocyte cell biology. Some brown adipocyte cells, now termed “classical” brown adipocytes, appear to derive from precursor cells common to skeletal muscle, whereas other brown adipocytes, referred to as beige or brite, appear to be more common to the white adipocyte cell lineage and, in fact, may be interspersed in white adipose tissue depots [16,17]. Whereas all types of brown adipocytes express *UCP1*, *PPARGC1A* (also known as PGC-1α) and *PRDM16* (PR domain-containing 16) genes, common markers of differentiation and thermogenic function, the expression levels of specific marker genes, such as *ZIC1* (zinc finger protein of the cerebellum 1) for classical brown adipocytes allow the distinction of both types of brown adipocytes [18]. Human brown fat in adults (i.e., that present mainly in the cervical and sub-scapular region) was initially considered to be composed mainly of beige/brite cells. However, recent data suggests that there are also classical brown adipocyte-enriched areas in adult human thermogenic adipose tissue [17,19–21].

Although lipoatrophic adipose tissue in HIV-1–associated lipodystrophy has been thoroughly studied [22–24], why some patients develop such opposing, hypertrophic events also in the subcutaneous area remains a mystery, and no clear-cut indicators of specific antiretroviral patterns or other mechanistic determinants have been identified. Moreover, it is unknown whether the brown fat-like phenotype appears only in BH (i.e., at anatomical areas close to those where brown fat is prevalent in adult humans) or is a general phenomenon of HIV-1–associated lipomatosis everywhere in the body.

In the present study, we sought to identify the main molecular events associated with the hypertrophic distortions that occur in adipose tissue in HIV-1 patients. To this end, we systematically characterized the molecular signature of lipomatosis in these patients by analyzing lipomatous samples at distinct anatomical regions.

## Materials and Methods

### Patients

HIV-1-infected patients were eligible if they had either lipomatous dorso-cervical enlargement (BH) or lipomas from different anatomical locations (non-BH) that required surgical removal. Subjects who were hospitalized or had a frank cognitive impairment on enrollment were not eligible. Patients with opportunistic infections, acute hepatitis, liver insufficiency, neoplasms or fever of undetermined origin were also excluded from the study. At the time of study enrollment no patients were using any other drugs known to influence glucose metabolism or fat distribution, such as anabolic hormones or systemic corticosteroids, recombinant human growth hormone, or appetite stimulants. Informed consent was obtained from the patients upon enrollment. Biopsies of BH adipose tissue from 10 patients were analyzed upon surgical extraction. These samples were compared to non-BH lipomas removed from different anatomical locations (two pubic, two sub-maxillary, one arm, three abdominal) in eight HIV-1–infected, HAART-treated patients, as well as samples from 10 age-matched, healthy controls obtained on occasion of minor dermatological surgical procedures from subcutaneous adipose tissue at anatomical sites comparable to those in lipomatous samples, i.e., abdominal and upper (dorsal and double chin) areas. To be eligible, controls did not have to meet any of the exclusion criteria used for patients.

Demographic values, anti-retroviral treatment data and circulating parameters are shown in Table 1. Age and body mass index (BMI) in patients and healthy controls were similar. Both patient groups had higher levels of serum triglycerides and cholesterol than controls. There were not significant differences in the cumulative time of treatment with the distinct types of anti-retroviral drugs (nucleoside analog reverse transcriptase inhibitors, NRTIs; non-nucleoside reverse transcriptase inhibitors, NNRTIs; protease inhibitors, PIs) between the two patient groups.

**Table 1:** Demographic values, treatment data and biochemical parameters in controls and HIV-1-infected patients bearing BH and NBL lipomas.

		HIV-1-infected patients	
	Control (n = 10)	Buffalo hump (n = 10)	Non-buffalo hump lipomas (n = 8)
**Age (years)**	57.4 ± 3.6	55.8 ± 1.4	54.2 ± 2.0
**Sex (n)**	6	7	5
**BMI (kg·m** ^**-2**^ **)**	26.1 ± 1.2	25.6 ± 2.6	25.3 ± 1.6
**Glucose (mmol·L** ^**-1**^ **)**	3.2 ± 0.3	3.7 ± 0.1	4.5 ± 0.8
**Triglycerides (mmol·L** ^**-1**^ **)**	1.2 ± 0.3	3.9 ± 0.5*	3.1 ± 0.6*
**Cholesterol (mmol·L** ^**-1**^ **)**	3.1 ± 0.6	5.6 ± 0.1*	6.2 ± 0.4*
**Clinical lipodystrophy**	No	Yes	Yes
**Time since HIV-1 infection (months)**	-	167 ± 18	177 ± 24
**Cumulative time (months) on:**			
**NRTIs**	-	140 ± 22	152 ± 20
**NNRTIs**	-	25 ± 2	32 ± 9
**PIs**	-	30 ± 12	38 ± 16
**CD4+ lymphocytes (cells·μL** ^**-1**^ **)**	-	549 ± 74	445 ± 84

Values are expressed as means ± SEM. Statistical differences between controls and both groups of lipodystrophic patients are shown as * whenever significant (p<0.05).

### Tissue processing, RNA and DNA extraction

All extractions were performed with the written informed consent of patients, and were approved by the Hospital de la Santa Creu i Sant Pau ethics committee. Once extracted, adipose tissue biopsies were immediately frozen in liquid nitrogen and stored at -80°C. For RNA transcript analysis, frozen samples were fragmented by mechanical disruption and then homogenized in RA-1 buffer (Macherey-Nagel, Düren, Germany) supplemented with 10% β-mercaptoethanol. RNA was immediately isolated from the homogenate using a column affinity-based methodology (NucleoSpin^®^ RNA II; Macherey-Nagel). DNA was separated from the tissue homogenate as a part of the protocol used for RNA extraction, dissolved in 70% ethanol, and purified by phenol/chloroform extraction.

### Gene expression analysis

cDNA was synthesized from 0.5 μg of total RNA using MultiScribe reverse transcriptase and random hexamer primers (Applied Biosystems, Foster City, CA, USA). mRNA expression levels were determined by quantitative real-time reverse transcription-polymerase chain reaction (qRT-PCR) using TaqMan reagents and an Applied Biosystems 7500 Fast Real-Time PCR System (Applied Biosystems). Real-time PCR was performed in a final volume of 20 μL using TaqMan Universal PCR Master Mix, No AmpErase UNG reagent, and the following specific primer probes (TaqMan Gene Expression Assays; Applied Biosystems) as detailed in S1 Table. Cytochrome *c* oxidase subunit II (*MT-CO2*) mRNA was quantified using custom-designed primers (forward, 5’-CAA ACC ACT TTC ACC GCT ACA C-3’; reverse, 5’-GAC GAT GGG CAT GAA ACT GT-3’) and FAM-labeled probe (5’-AAA TCT GTG GAG CAA ACC-3’) obtained from Applied Biosystems (Custom TaqMan Gene Expression Assays). mRNA levels of the genes of interest, normalized to that of the reference control (18S ribosomal RNA), were calculated using the comparative 2^-ΔΔC^_T_ method.

### Protein expression analysis

For Western blot analysis of protein expression, fragments of previously disrupted adipose tissue were homogenized in cold buffer consisting of 10 mmol·L^-1^ HEPES (pH 7.5), 5 mmol·L^-1^ ethylenediamine tetraacetic acid, 5 mmol·L^-1^ dithiothreitol, and 5 mmol·L^-1^ MgCl_2_ supplemented with protease inhibitor (Complete-mini; Roche, Sant Cugat del Vallés, Spain). The protein concentration in each sample was determined by the Bradford method, with detection of absorbance at 595 nm absorbance using a Shimadzu UV 160-A spectrophotometer. Homogenates containing equal amounts of protein (40 μg) were brought to a total volume of 35 μL with bidistilled water, mixed with 1/5 volume loading buffer (10% sodium dodecyl sulfate [SDS], 10% β-mercaptoethanol, 0.5 M Tris pH 6.8, 50% glycerol, 0.5% bromophenol blue) and incubated at 95°C for 5 min. Proteins in samples were then resolved on SDS-containing 10%–12% polyacrylamide gels and transferred to Immobilon-P nitrocellulose membranes (Millipore, Billerica, MA, USA) using a Mini Trans-Blot kit (Bio-Rad Laboratories, Hercules, CA, USA). Blots were incubated with primary antibodies against PPARγ (sc-1984X), LPL (sc-73646), GLUT4 (sc-7938), PCNA (sc-25280), adiponectin (sc-26497) and lamin A/lamin C/prelamin A (sc-6215), obtained from Santa Cruz Biotechnology (Santa Cruz, CA, USA); β2-microglobulin (P0163), obtained from Dako Cytomation (Glostrup, Denmark); cytochrome *c* oxidase subunit II (12C4), obtained from Molecular Probes (Eugene, OR, USA); p53 (2570S) from Cell Signaling (Danvers, MA, USA) or β-actin (A5441), obtained from Sigma Chemical Co. (St Louis, MO, USA). After incubating with primary antibodies, blots were incubated with horseradish peroxidase (HRP)-conjugated goat anti-mouse IgG (BioRad), donkey anti-rabbit IgG (Jackson Immunoresearch, West Grove, PA, USA) or rabbit anti-goat IgG (Sigma), as appropriate. Immunoreactive proteins were detected using a Millipore Immobilon Western chemiluminescent HRP system (Millipore) and a Fujifilm ImageRecorder LAS-3000 detection device (Fujifilm, Tokyo, Japan). The optical density of each band was quantified using the MultiGauge 3.0 software suite (Fujifilm).

### mtDNA quantification

Total DNA isolated from tissue samples was purified by phenol/chloroform extraction, as indicated above, and the relative abundance of mtDNA was assessed by real-time PCR as previously described [7]. mtDNA levels were expressed as the amount of the mitochondrial gene *MT-CYB* (cytochrome B) relative to the nuclear, intronless, single-copy gene *CEBPA* (CCAAT/enhancer binding protein alpha). The TaqMan probes used to determine *MT-CYB* and *CEBPA* expression are shown in S1 Table.

### Telomere relative length quantification

Telomere length was assessed by real-time PCR essentially as described previously [25]. Briefly, PCR was performed using 35 ng of total DNA (PCR template) isolated from tissue homogenates in a reaction containing 10 μL of 2x SYBR Green Master Mix reagent (Applied Biosystems) and 100 nM of specific primers (Sigma) for telomere repeats (Tel) or the single-copy housekeeping gene *36B4* (acidic ribosomal phosphoprotein P0) in a final volume of 20 μL. The following primer pairs were used: Tel, 5’-CGG TTT GTT TGG GTT TGG GTT TGG GTT TGG GTT TGG GTT-3’ (forward) and 5’-GGC TTG CCT TAC CCT TAC CCT TAC CCT TAC CCT TAC CCT-3’ (reverse); and 36B4, 5’-GCA AGT GGG AAG GTG TAA TCC-3’ (forward) and 5’-ATT CTA TCA TCA ACG GGT ACA A-3’ (reverse). C_T_ values were obtained for both Tel and 36B4 in each sample. Relative telomere abundance was quantified as the Tel/36B4 ratio using the comparative 2^-ΔΔC^_T_ method.

### Statistical analysis

Results are expressed as means ± SEM. Statistical comparisons between groups were performed using a non-parametric analysis (Mann-Whitney U test). Statistical differences were considered significant for p-values < 0.05.

## Results

**Fig 1:**
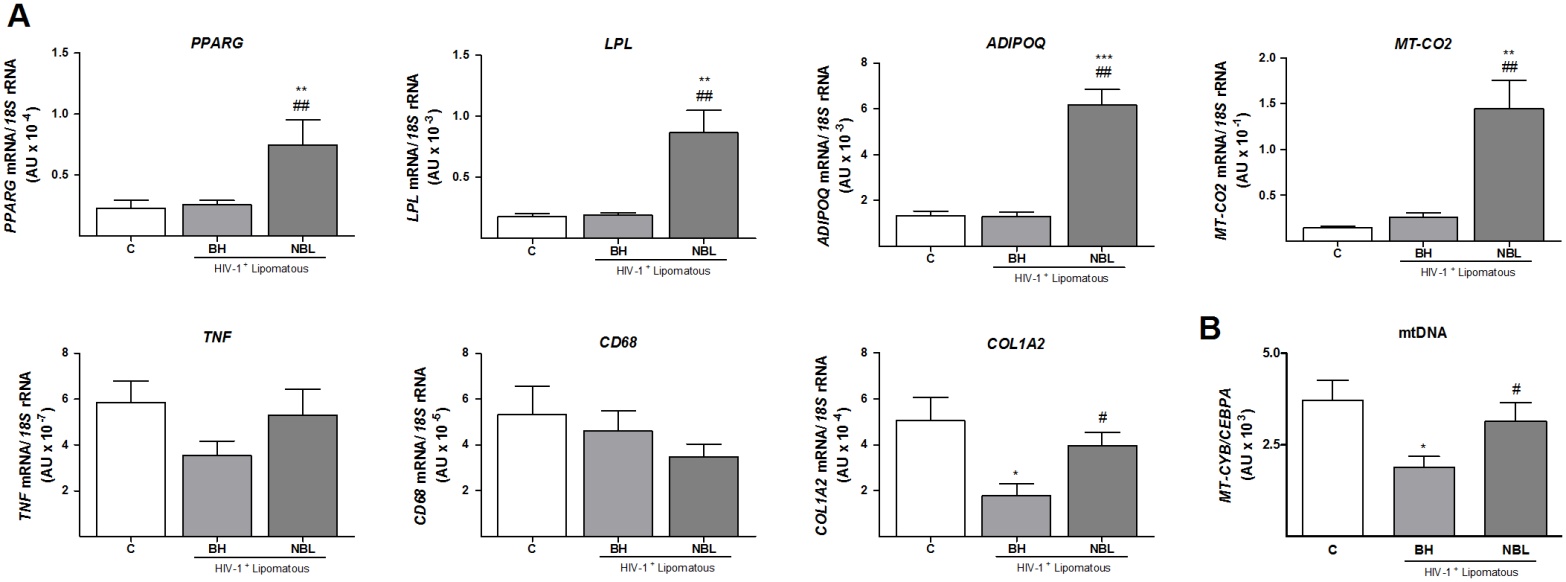
Expression of mRNA for various marker genes and mtDNA levels in BH and non-BH lipomas from HIV-1–infected, HAART-treated patients and healthy control subcutaneous adipose tissue. **A)** Relative mRNA levels of the indicated markers of adipogenesis and adipocyte function, mitochondrial function, inflammation, and fibrosis were determined by qRT-PCR. Means ± SEM, expressed as ratios relative to 18S rRNA, are shown for each target mRNA. B) mtDNA data are presented as means ± SEM, expressed as a ratio units between the mitochondrial gene *MT CYB* and the single-copy nuclear gene *CEBPA* levels (*p<0.05, lipomas vs. healthy control; #p<0.05, NBL vs. BH). (*p<0.05, **p<0.01 and ***p<0.001, lipoma vs. healthy subcutaneous adipose tissue; #p<0.05, ##p<0.01, NBL vs BH). Means correspond to 10 (C, BH) and 8 (NBL) samples.

The levels of transcripts for marker genes related to adipogenesis and adipose function (PPAR-γ, LPL and AdipoQ), inflammation (TNFα), macrophage infiltration (CD68), mitochondrial toxicity (MT-CO2), and fibrosis (COL1A2) were determined in lipomatous and healthy control adipose tissues (Fig 1). Lipomatous adipose tissue data were analyzed separately for BH samples and non-BH lipomatous tissue (NBL), as described in Materials and Methods.

Transcript levels for adipogenesis marker genes, including peroxisome proliferator-activated receptor (PPAR)-γ, the master regulator of adipose differentiation, LPL (lipoprotein lipase), the enzyme responsible for fatty acid uptake and fat accretion in adipose tissue, and adiponectin, a main adipokine, were unaltered in BH compared with healthy subcutaneous adipose tissue. However, each of these three gene transcripts was systematically up-regulated in NBL from HIV-1–infected patients. Tumor necrosis factor (TNF)-α, a marker of inflammation, and CD68, a macrophage infiltration marker, were not altered in any of the adipose tissue sample groups analyzed. Expression of the mtDNA-encoded transcript *MT-CO2* was altered only in NBL samples, where it was significantly increased. Expression of the fibrosis marker *COL1A2* (collagen type I alpha 2) trended lower in BH and NBL samples relative to controls, but only the reduction in BH reached statistical significance.

Given that mitochondrial toxicity, specifically mtDNA depletion, is a relevant feature of co-morbidities present in HIV-1–infected patients undergoing treatment, we next quantified mtDNA levels. These analyses revealed that mtDNA levels were significantly reduced in BH samples relative to healthy controls, but were unaltered in NBL (Fig 1B).

**Fig 2:**
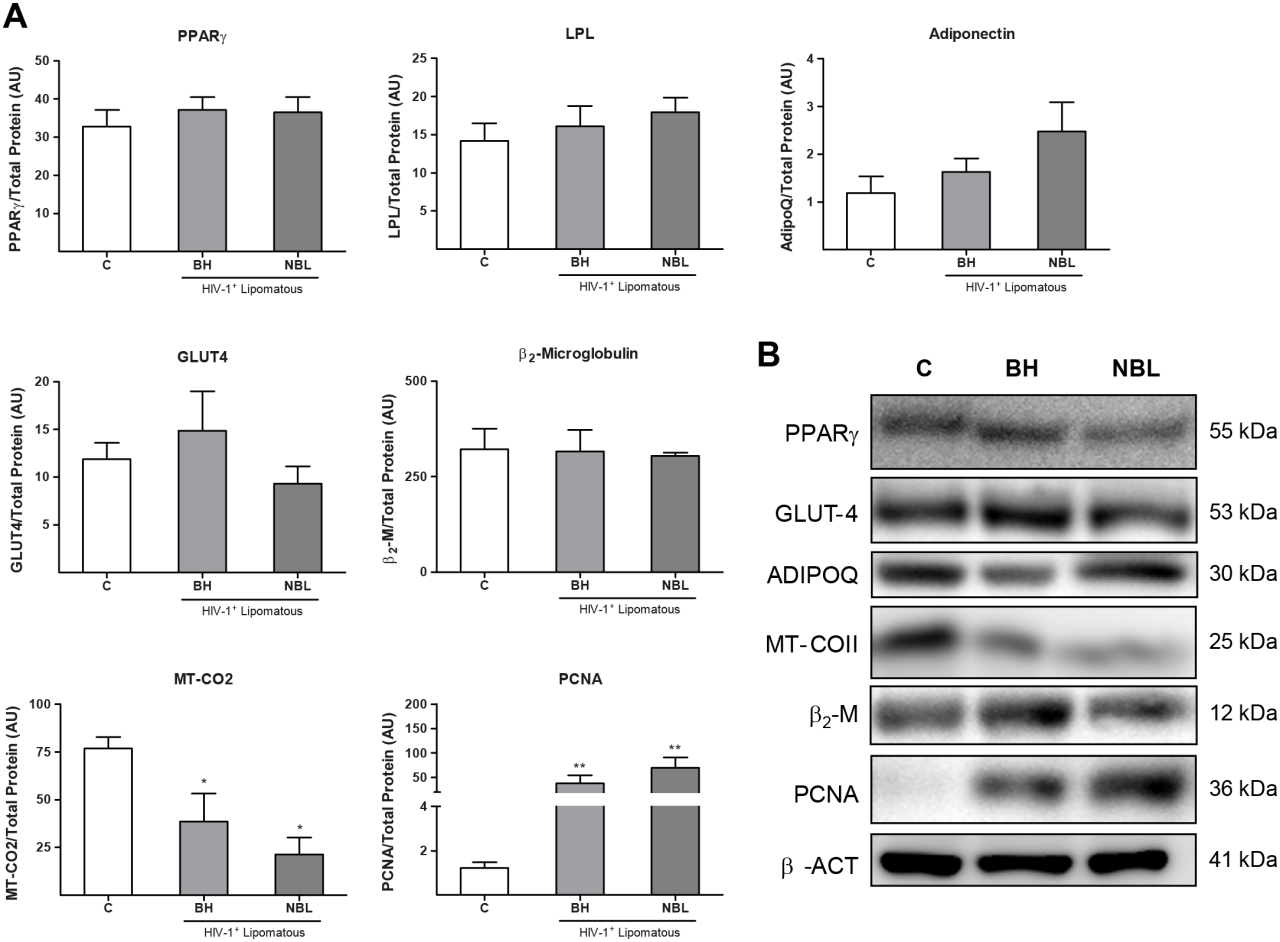
Expression of protein for various marker genes in BH and non-BH lipomas from HIV-1–infected, HAART-treated patients and healthy control subcutaneous adipose tissue. **A)** Relative protein levels of the indicated markers of adipogenesis and adipocyte function, mitochondrial function, inflammation, and cell proliferation were determined by densitometric analysis of Western blots. Means ± SEM of 10 (C, BH) and 8 (NBL) samples expressed as ratios of the optical density of each band corrected for total protein level, are shown for each protein (*p<0.05, **p<0.01 and, lipomas vs. healthy subcutaneous adipose tissue; #p<0.05, ##p<0.01 and ###p<0.001, NBL vs. BH). **B)** Representative Western blot bands for selected genes of each functional group in panel A for controls (C), BH, and NBL. Each sample corresponds to an individual from each group. β-actin was used a loading control. The molecular weight of each specific immunoreactive signal is shown at right. ADIPOQ, adiponectin; MT_COII, mitochondrial DNA-encoded subunit II of cytochrome c oxidase.

We next extended our study to an analysis of marker expression at the protein level (Fig 2A and 2B). Unlike transcripts, protein levels of PPARγ, LPL and adiponectin as well as those of the glucose transporter GLUT4, another protein associated with adipogenesis, were not significantly different between BH or NBL samples and controls. The abundance of β_2_-microglobulin, an indicator of inflammation, was also unaltered. Levels of the mtDNA-encoded protein MT-CO2 were significantly reduced relative to controls to a similar extent in both BH and NBL samples. An analysis of the expression of the proliferation marker, PCNA [26], showed a substantial increase in PCNA protein levels in BH and NBL relative to healthy subcutaneous adipose tissue, with the extent of the increase being similar for the two types of lipomatous tissue.

Considering the differences found, and taking into account the distinct anatomical location of NBL samples, we analyzed the gene expression and protein data by separately pooling data from samples taken from upper and lower body areas (using the waist as the dividing line). As shown in S2 Table, the only significant anatomical site-dependent changes observed were minor differences for *MT-CO2* and adiponectin expression in both NBL patients and healthy controls.

**Fig 3:**
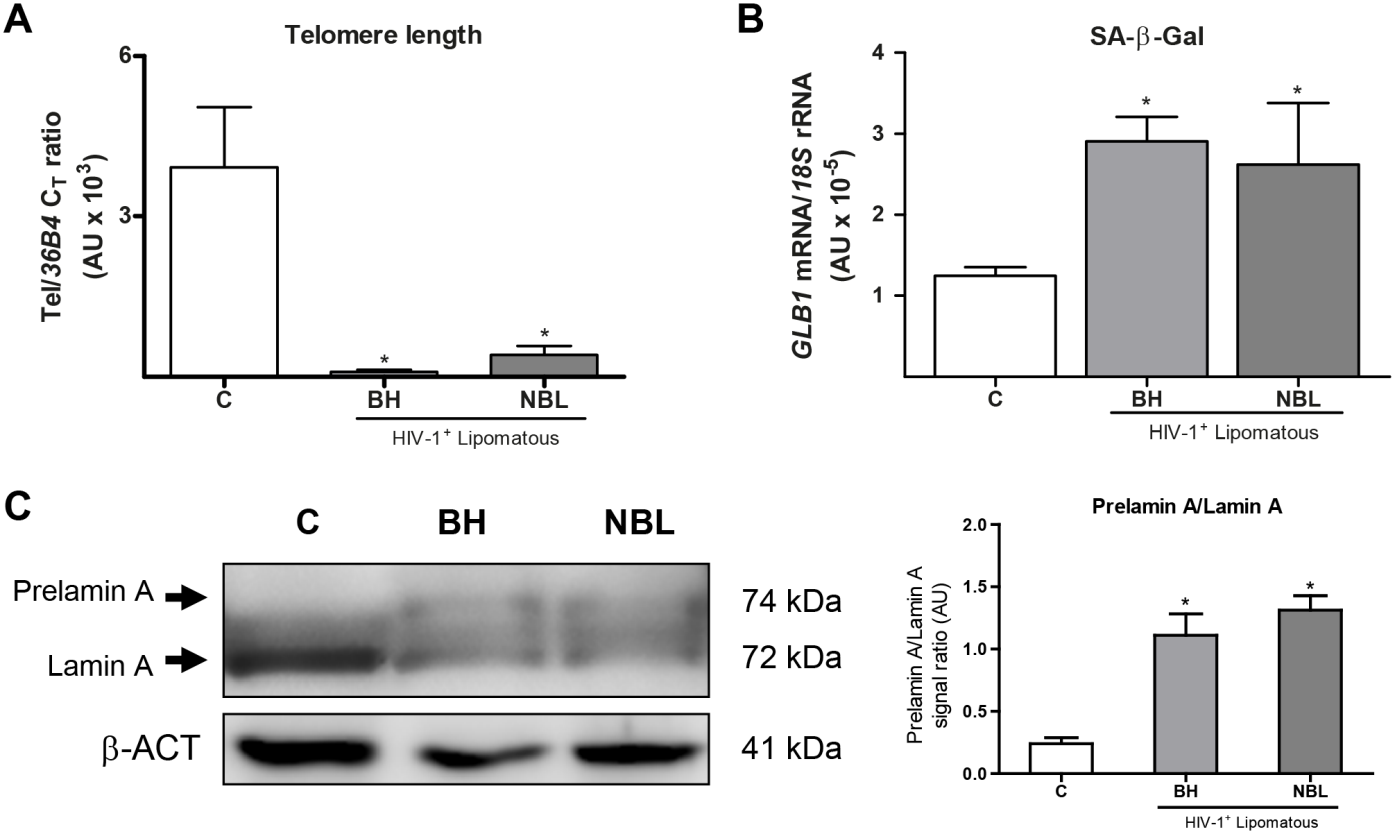
Relative telomere length, expression of *GLB1* (SA-β galactosidase) and prelamin A/lamin A ratio in adipose tissue from BH and NBL lipomas from HIV-1–-infected, HAART-treated individuals and in subcutaneous adipose tissue from healthy controls. **A)** Relative telomere lengths are presented as means ± SEM, expressed as a ratio of arbitrary fluorescence units for telomere repeats (Tel) to the single-copy nuclear gene 36B4 (*p<0.05, lipomas vs. healthy controls). B) Relative mRNA levels of SA-β-Galactosidase. Means ± SEM, expressed as ratios relative to 18S rRNA, are shown. C) Prelamin A/lamin A ratio are presented as means ± SEM of the relative densitometric analysis of western blots (right), representative western blot bands for lamin A and prelamin A are shown (left). The bands for both processed lamin and unprocessed prelamin are indicated with arrows. Means correspond to 10 (C, BH) and 8 (NBL) samples.

Accelerated ageing is often considered to be associated with co-morbidities that appear in HIV-1 patients, and telomere length in adipose tissue has been proposed as a marker of senescence and ageing-related phenomena that occur in fat under pathological conditions such as obesity [27]. We found that telomere length was profoundly decreased in both BH and NBL relative to healthy controls, with no differences between the two types of lipomatous tissue (Fig 3A). Notably, telomere length in lipoatrophic, non-lipomatous, adipose tissue from HIV-1–infected patients was not altered compared with that in healthy controls (data not shown). Determination of the expression of the *GLB1* gene, encoding SA-β-Gal [28], a marker of senescence, indicated a significant increase in both BH and NBL relative to controls (Fig 3B). We also measured p53 levels as a further marker of senescence but, in this case, no significant differences were found (data not shown).

Several partial lipodystrophy syndromes of genetic origin, sometimes associated with local lipomatous phenomena, are caused by alterations in the synthesis of lamin A protein that results in accumulation of the unprocessed prelamin A form in lipomatous tissue [29]. This phenomenon has been related to accelerated ageing [30]. We detected this high-molecular-weight, unprocessed form of prelamin A in BH and NBL samples, but found only weak expression in control adipose tissue, thus the prelamin A/lamin A ratio was increased in both BH and NBL relative to healthy control adipose tissue. (Fig 3C).

**Fig 4:**
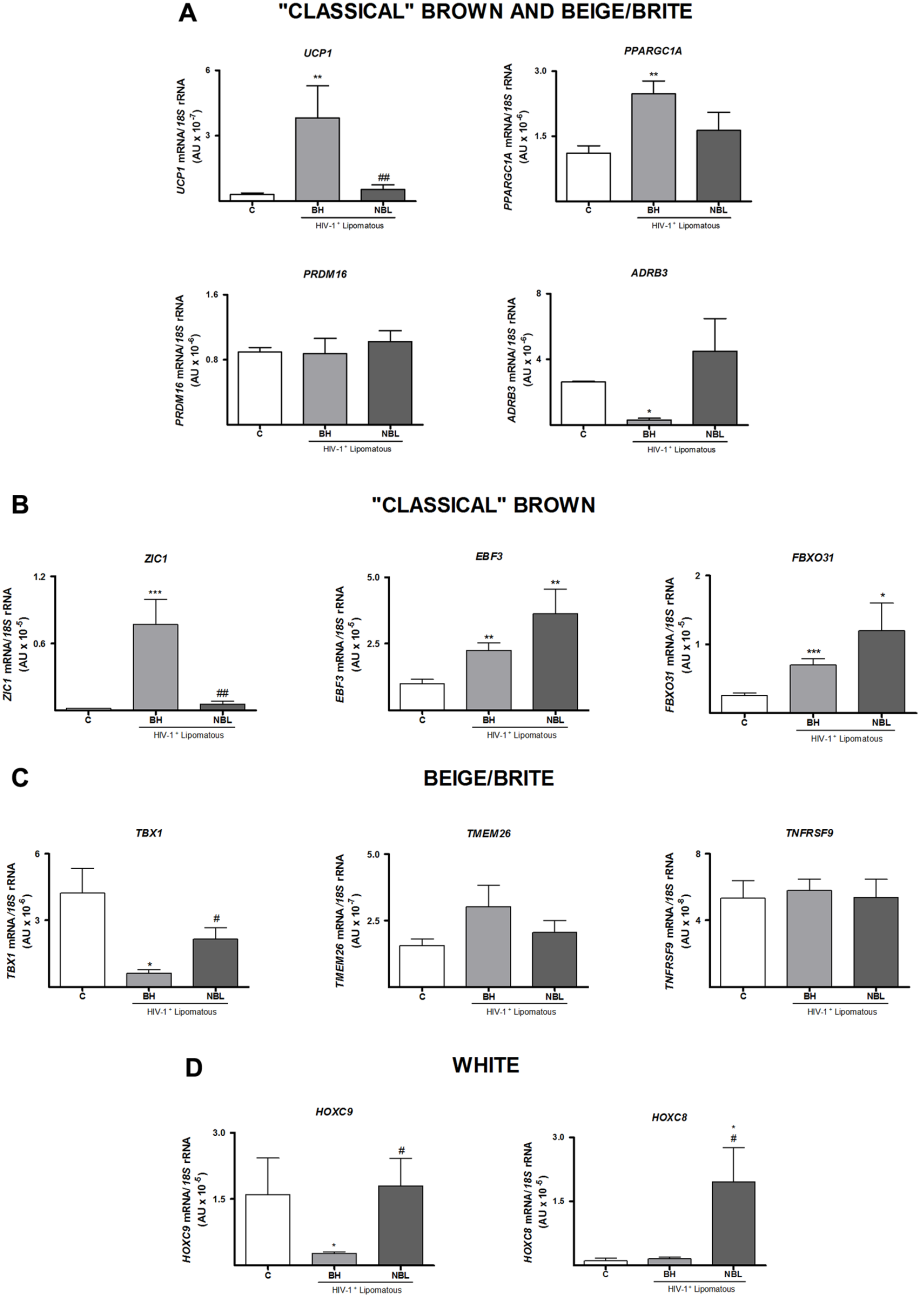
Expression of mRNA for brown adipocyte-associated genes and specific lineage markers of different classes of adipocytes in adipose tissue from BH and NBL lipomas from HIV-1–infected, HAART-treated individuals and in subcutaneous adipose tissue from healthy controls (C). Relative mRNA levels were determined by qRT-PCR. Means ± SEM of 10 (C, BH) and 8 (NBL) samples, expressed as ratios relative to 18S rRNA, are shown for each target mRNA (*p<0.05, **p<0.01 and ***p<0.001, lipomas vs. healthy subcutaneous adipose tissue; #p<0.05, ##p<0.01 and ###p<0.001, NBL vs. BH lipomas). **A)** Brown adipocyte-associated genes. **B-D)** Specific putative lineage markers of different types of adipocytes.

Finally, we analyzed the expression of brown-versus-white adipose tissue marker genes (Fig 4A). Expression of *UCP1* and *PPARGC1A* mRNA was significantly higher in BH, but not NBL, compared with healthy control adipose tissue, whereas *PRDM16* transcript levels were unchanged. Expression of the β_3_-adrenergic receptor, on the other hand, was dramatically repressed in BH, but not in NBL. We further analyzed the expression of marker genes recently proposed to distinguish between classical or developmentally programmed brown adipose tissue and inducible or beige/brite brown adipose tissue [31,32] (Fig 4B–4D). *ZIC1* mRNA, which was highly expressed in BH, showed negligible and very low expression in control adipose tissue and NBL, respectively. In contrast, *EBF3* (early B-cell factor 3) and *FBXO31* (F-box only protein 31), two other marker genes proposed to distinguish between the two types of brown fat, were upregulated in common in BH and NBL compared with controls. An analysis genes proposed in some studies to be markers of the beige/brite type of fat showed that expression of *TBX1* (T-box 1) was decreased in both enlarged BH and NBL sites compared with control fat, whereas *TMEM26* and *TNFRSF9/CD137* (tumor necrosis factor receptor superfamily, member 9) expression were not different among adipose tissues. It has been proposed that expression of *HOXC8* (homeobox C8) and *HOXC9* (homeobox C9) genes is preferentially associated with the white adipocyte phenotype relative to either the classical or beige/brite brown phenotype. We found that *HOXC9* expression was dramatically reduced in BH but unaltered in NBL relative to controls, whereas *HOXC8* was significantly increased in NBL.

## Discussion

Dorso-cervical adipose accumulation, often referred as buffalo hump, is an alteration that occurs with a certain frequency in HIV-1 patients undergoing antiretroviral treatment, but attempts to associate it with specific patterns of treatment or other variables have been unsuccessful. There have been recent reports of the appearance of BH even in patients undergoing newer drug treatment regimens (e.g., raltegravir-based) with low toxicity towards adipose function and metabolism [33]. In addition to BH, adipose enlargements randomly occur in patients at other sites of subcutaneous adipose tissue, with the highest frequency observed in the neck [5] and pubic [4] regions. Previous studies have shown that expression of adipogenesis and inflammation marker genes is unaltered in BH in HIV-1–infected, HAART-treated patients; this contrasts with the repressed adipogenesis and enhanced local inflammation observed in lipoatrophic subcutaneous fat [7]. We found that non-BH enlarged adipose depots apart from BH presented similar patterns of unaltered adipogenesis and inflammation, at least at the protein level. This finding confirms that hypertrophy of subcutaneous fat is associated with protection from the induction of pro-inflammatory responses that are known to take place at sites prone to lipoatrophy in HIV-1 patients. A feature of NBL that distinguishes it from BH is that protein markers of adipogenesis (LPL, PPARγ, adiponectin) are unchanged despite enhanced expression of the corresponding transcripts. The mechanisms underlying this discordance are unknown, but the fact that post-transcriptional and post-translational mechanisms are known to be relevant for determining LPL and PPAR-γ protein levels in adipose tissues should be taken into account [34,35]. Discordances between changes in adiponectin transcript and protein levels occur in subcutaneous adipose tissue from HIV-1-infected patients [36,37]. Moreover, as a secreted protein, adiponectin steady-state levels in cells may not change despite concerted alterations in synthesis and output, a fact that may also help explain this observation.

Indeed, our current findings support the lipomatous character of these other subcutaneous adipose tissue enlargements that appear locally in HIV-1 patients (i.e., pubic area, circumferential neck), as previously reported for BH [7]. The increased expression of PCNA, a bona fide marker of cell proliferation, confirms the activated cell-cycle status in the distinct, enlarged adipose tissue depots analyzed here. This means that lipid accumulation and hypertrophy alone are not sufficient to explain the adipose enlargement, suggesting that hyperplasia of adipose tissue contributes to adipose depot overgrowth, both in BH and at distinct anatomical sites. Fibrosis, a feature usually found in rapidly proliferating adipose depots that leads to inflammation [38], was not increased, at least not at the transcriptional level. Notably, we found a dramatic reduction in DNA telomere length in both BH and non-BH enlarged adipose depots relative to control healthy adipose tissue, finding that strongly supports the lipomatous character of these adipose sites. Shortening of telomeres is a consequence of tissues having experienced high rates of cell replication, as has previously been reported for adipose depots in obese patients [27,39]. As a non-malignant tumorigenic process, lipomatosis maintains telomere shortening with cell division [40]; this contrasts with malignant tumorigenesis, which evades telomere shorting through activation of telomerase [39,41]. To our knowledge, our current findings represent the first data on telomere length as a marker of cellular behavior in fat depots from HIV-1–infected patients.

The common lipomatous character of BH and non-BH adipose enlargements is also evidenced by the accumulation of the toxic, non-processed form of lamin A, a phenomenon previously reported to take place not only in genetically-determined lipomatosis [29,42] but also in BH from HIV-1–infected, HAART-treated patients [9], as well as in the lipoatrophic areas of these individuals. Our results, taken together with previous observations [43,44], suggest that lipomatosis in HIV-1 patients reflects an increased aging phenotype, as evidenced by prelamin A accumulation, telomere length reduction and enhanced expression of SA-β-Galactosidase. In contrast to lipoatrophic areas, hyperproliferative lipomas would therefore compensate for cell death with accelerated precursor cell turnover, thus growing in size.

Our current findings confirm previous reports indicating partial acquisition of a brown adipose tissue molecular signature in BH. Our demonstration of increased expression of UCP1 is in accord with previous findings [7–9]. The partial acquisition of a brown adipose tissue phenotype is further underscored by the strong repression of β_3_-adrenoreceptor mRNA expression—a common finding in thermogenically activated brown adipose tissue in experimental models [45]. However, non-BH lipomatous sites did not show a remarkable expression of UCP1. Even when non-BH samples from circumferential neck enlarged adipose tissue close to sites where physiological brown adipose tissue is known to be present were analyzed separately, UCP1 expression was negligible. These findings indicate that the acquisition of a partial brown fat-type molecular signature is a specific feature of BH that is not shared by lipomas at other anatomical sites in HIV-1 patients.

This study represents the first use of marker genes proposed to allow the distinction between beige/brite and classical brown fat to characterize the brown fat-like features of BH. Our finding of very high expression of *ZIC1*, possibly the most specific, and best accepted, marker of classical, non-beige/brite, brown adipocytes [19–21,46], indicates that abnormal classical brown adipogenesis underlies the BH phenotype.

The suppressed expression of *HOXC9*, a proposed marker of the white-versus-brown phenotype [19,20], specifically in BH further supports the shift from white to brown adipogenesis in BH. The information obtained regarding a putative beige/brite phenotype was less conclusive, as only some increase in NBL versus BH expression was found. In fact, the determination of classical brown-versus-beige/brite phenotype made on the basis of marker gene expression analysis should be considered with caution, considering how recently these markers, the lack of unanimous agreement on their use and scarce validation in humans tissue of data obtained in rodents [46–48]. In this context, some studies have reported that *TMEM26* is preferentially found in human BAT [17,32] whereas others have reported no differences between BAT and WAT [19,20]. The value of *TNFRSF9 (CD137)* as bona-fide beige marker has also been questioned, and a recent comprehensive compilation of available data suggested that, whereas *ZIC1* is emerging as a bona-fide marker of “classical” BAT in humans, the validity of beige/briter markers in human adipose tissues is less compelling [46].

Overall, our findings strongly support the conclusion that a brown fat-like phenotype related to the classical brown adipocyte cell lineage is specifically induced in HIV-1–associated BH but not in lipomas at other anatomical sites. Recent reports have claimed that adipocytes of the classical brown type are present in human adults [19,20]; thus, it is likely that a specific distortion of this cell type elicited by HIV-1 infection and/or antiretroviral treatment underlies the etiopathology of BH lipomatosis.

The present study has obvious limitation: the relative scarcity of samples in this type of studies, the anatomical variability of NBL location, the impossibility to perform the study in distinct lipomas from the same indivdual, and the single molecular approach followed. We were nonetheless able to conclude that BH and adipose enlargements that occur at different anatomical sites in HIV-1–infected, HAART-treated patients share a similar lipomatous character and the absence of a local pro-inflammatory status. However, only BH exhibited a distortion in the brown-to-white molecular signature that appears to involve the classical brown adipocyte cell lineage. Overall, the present data provide evidence that HIV-1 infection and antiretroviral toxicity-mediated alterations in adipose tissue plasticity may differentially impact distinct anatomical sites according to a distinct molecular signature of affected tissue areas. Although our data allow few conclusions to be reached regarding prevention or treatment of this alteration, they suggest that proposals to promote brown adipose tissue activation as a tool for improving the metabolic syndrome, especially as it applies to HIV-1–infected patients, should therefore be considered with caution in the future.

## Supporting Information

S1 Table Reference numbers for the TaqMan probes used in the gene expression experiments conducted in this study. (DOC)

S2 Table mRNA and protein levels of adipogenesis and adipocyte function, mitochondrial function, inflammation, fibrosis, proliferation and brown-versus-white marker genes of subcutaneous adipose tissue from the upper body and lower body region of healthy controls (C) and non-buffalo lipomas (NBL). (DOC)
