# Supplementary material for: The Molecular Signature of HIV-1-Associated Lipomatosis Reveals Differential Involvement of Brown and Beige/Brite Adipocyte Cell Lineages
Source: PLoS One. 2015 Aug 25;10(8):e0136571. doi: 10.1371/journal.pone.0136571 (PMC4549259; doi:10.1371/journal.pone.0136571)
Supplement: S1 Table — (DOC) [file pone.0136571.s001.doc]

| **Gene** | **TaqMan probe reference number** |
| --- | --- |
| *PPARG* | Hs00234592_m1 |
| *LPL* | Hs00173425_m1 |
| *ADIPOQ* | Hs00605917_m1 |
| *TNF* | Hs00174128_m1 |
| *CD68* | Hs00154355_m1 |
| *COL1A2* | Hs00164099_m1 |
| *MT-CYB* | Hs02596867_s1 |
| *CEBPA* | Hs00269972_s1 |
| *UCP1* | Hs00222453_m1 |
| *PPARGC1A* | Hs00173304_m1 |
| *PRDM16* | Hs00223161_m1 |
| *ADRB3* | Hs00609046_m1 |
| *ZIC1* | Hs00602749_m1 |
| *EBF3* | Hs00406051_m1 |
| *FBXO31* | Hs00375551_m1 |
| *TBX1* | Hs00962556_m1 |
| *TMEM26* | Hs00415619_m1 |
| *TNFRSF9* | Hs00155512_m1 |
| *HOXC8* | Hs00224073_m1 |
| *HOXC9* | Hs00396786_m1 |
| *GLB1* | Hs01035168_m1 |
| *TP53* | Hs00153349_m1 |
| *18S* | Hs99999901_m1 |

**S1 Table: Reference numbers for the TaqMan probes used in the gene expression experiments conducted in this paper.**
