# Supplementary material for: The Molecular Signature of HIV-1-Associated Lipomatosis Reveals Differential Involvement of Brown and Beige/Brite Adipocyte Cell Lineages
Source: PLoS One. 2015 Aug 25;10(8):e0136571. doi: 10.1371/journal.pone.0136571 (PMC4549259; doi:10.1371/journal.pone.0136571)
Supplement: S2 Table — (DOC) [file pone.0136571.s002.doc]

| **Gene** | **Upper body C**  **(n = 5)** | **Lower body C**  **(n = 5)** | **Upper body NBL**  **(n = 3)** | **Lower body NBL**  **(n = 5)** |
| --- | --- | --- | --- | --- |
| *PPARG* | 2.9·10^-5^ ± 9.6·10^-6^ | 3.2·10^-5^ ± 5.7·10^-6^ | 5.5·10^-5^ ± 3.5·10^-6^ | 7.2·10^-5^ ± 2.1·10^-6^ |
| *LPL* | 1.6·10^-4^ ± 5.1·10^-5^ | 1.8·10^-4^ ± 1.7·10^-5^ | 5.4·10^-4^ ± 1.8·10^-5^ | 7.8·10^-4^ ± 1.2·10^-4^ |
| *ADIPOQ** | 6.1·10^-4^ ± 2.4·10^-4^ | 1.7·10^-3^ ± 2.0·10^-4^ | 2.7·10^-3^ ± 8.8·10^-4^ | 6.2·10^-3^ ± 6.8·10^-4^ |
| *TNF* | 1.1·10^-6^ ± 1.2·10^-7^ | 4.7·10^-7^ ± 8.5·10^-8^ | 4.9·10^-7^ ± 1.9·10^-7^ | 5.3·10^-7^ ± 1.2·10^-7^ |
| *CD68* | 5.0·10^-5^ ± 1.4·10^-5^ | 4.2·10^-5^ ± 1.2·10^-5^ | 5.3·10^-5^ ± 1.4·10^-5^ | 3.8·10^-5^ ± 8.1·10^-6^ |
| *MT-COII*#* | 1.3·10^-2^ ± 2.3·10^-3^ | 6.0·10^-2^ ± 1.1·10^-3^ | 2.9·10^-2^ ± 2.2·10^-2^ | 1.4·10^-1^ ± 3.1·10^-2^ |
| *COL1A2* | 5.1·10^-4^ ± 1.2·10^-4^ | 5.0·10^-4^ ± 1.6·10^-4^ | 3.3·10^-4^ ± 4.8·10^-5^ | 3.7·10^-4^ ± 6.7·10^-5^ |
| *UCP1* | 3.1·10^-8^ ± 5.4·10^-9^ | 2.0·10^-8^ ± 1.4·10^-8^ | 3.9·10^-8^ ± 2.4·10^-8^ | 5.5·10^-8^ ± 3.3·10^-8^ |
| *PPARGC1A* | 1.4·10^-6^ ± 1.3·10^-7^ | 8.4·10^-7^ ± 6.5·10^-8^ | 1.4·10^-6^ ± 7.2·10^-7^ | 1.5·10^-6^ ± 2.1·10^-7^ |
| *PRDM16* | 8.3·10^-7^ ± 5.6·10^-8^ | 9.7·10^-7^ ± 8.5·10^-8^ | 1.3·10^-6^ ± 1.6·10^-7^ | 8.0·10^-7^ ± 3.5·10^-7^ |
| *ADRB3* | 2.7·10^-6^ ± 1.7·10^-7^ | 2.5·10^-6^ ± 4.8·10^-7^ | 2.1·10^-6^ ± 1.2·10^-6^ | 3.6·10^-6^ ± 1.7·10^-6^ |
| *ZIC1* | 2.2·10^-7^ ± 4.4·10^-8^ | 1.3·10^-7^ ± 6.2·10^-8^ | 1.0·10^-6^ ± 5.8·10^-7^ | 5.3·10^-8^ ± 2.9·10^-8^ |
| *EBF3* | 1.6·10^-5^ ± 7.2·10^-6^ | 2.0·10^-5^ ± 1.2·10^-5^ | 3.7·10^-5^ ± 2.8·10^-5^ | 3.9·10^-5^ ± 1.8·10^-5^ |
| *FBXO31* | 3.1·10^-6^ ± 1.1·10^-6^ | 1.7·10^-6^ ± 1.2·10^-6^ | 8.4·10^-6^ ± 4.7·10^-6^ | 1.4·10^-5^ ± 7.7·10^-6^ |
| *TBX1* | 2.2·10^-6^ ± 5.4·10^-7^ | 6.3·10^-7^ ± 1.2·10^-7^ | 2.8·10^-6^ ± 1.2·10^-6^ | 2.2·10^-6^ ± 7.3·10^-7^ |
| *TMEM26* | 2.7·10^-7^ ± 1.9·10^-8^ | 1.1·10^-7^ ± 7.6·10^-8^ | 1.7·10^-7^ ± 7.7·10^-8^ | 2.4·10^-7^ ± 1.4·10^-8^ |
| *TNFRSF9* | 5.1·10^-4^ ± 1.2·10^-4^ | 5.0·10^-4^ ± 1.6·10^-4^ | 5.5·10^-8^ ± 3.1·10^-8^ | 6.2·10^-8^ ± 3.8·10^-8^ |
| *HOXC9* | 8.1·10^-6^ ± 3.2·10^-6^ | 2.5·10^-5^ ± 2.6·10^-6^ | 1.5·10^-5^ ± 8.5·10^-6^ | 2.5·10^-5^ ± 9.6·10^-6^ |
| *HOXC8* | 4.1·10^-7^ ± 1.8·10^-7^ | 1.8·10^-6^ ± 6.6·10^-7^ | 1.6·10^-5^ ± 3.1·10^-6^ | 7.6·10^-5^ ± 2.7·10^-5^ |
| **Protein** |  |  |  |  |
| PPARγ | 29.7 ± 7.4 | 34.7 ± 5.9 | 37.6 ± 5.7 | 37.3 ± 4.7 |
| LPL | 12.9 ± 2.8 | 14.8 ± 3.5 | 20.1 ± 0.4 | 17.6 ± 2.3 |
| GLUT4 | 13.7 ± 3.0 | 11.0 ± 1.6 | 11.2 ± 0.7 | 9.7 ± 1.5 |
| ADIPOQ | 1.6 ± 0.1 | 1.3 ± 0.7 | 3.8 ± 0.8 | 2.1 ± 0.7 |
| B_2_M | 306.8 ± 68.8 | 335.3 ± 95.2 | 313.5 ± 20.6 | 301.0 ± 9.7 |
| MT-COII | 86.6 ± 1.1 | 70.2 ± 8.1 | 26.6 ± 9.5 | 23.7 ± 11.0 |
| PCNA | 1.3 ± 0.5 | 1.1 ± 0.2 | 38.8 ± 10.3 | 75.3 ± 17.6 |

**S2 Table: mRNA and protein levels of adipogenesis and adipocyte function, mitochondrial function, inflammation, fibrosis, proliferation and brown-versus-white marker genes of subcutaneous adipose tissue from the upper body and lower body region of healthy controls (C) and non-buffalo lipomas (NBL).** Means ± SEM are shown for each mRNA concentration (expressed as ratios relative to 18S rRNA) and for each western blot band quantification data for proteins (expressed as ratios of each band’s optical density corrected for total protein quantification). Differences were considered to be statistically significant when P<0.05. Statistical differences between upper and lower regions are shown as * for C or as # for NBL whenever significant.
